# A spatiotemporal U-Net++ deep learning framework for dengue risk mapping in Colombia

**DOI:** 10.1038/s41598-026-63542-8

**Published:** 2026-08-03

**Authors:** Daira Velandia, Javiera Contador, Juan Zamora, Diana Martínez, Débora Buendía, Ximena Collao, Rodrigo Salas

**Affiliations:** 1 Institute of Statistics, Universidad de Valparaíso, Valparaiso, Chile; 2 Center for Atmospheric Studies and Climate Change (CEACC), Universidad de Valparaíso, Valparaiso, Chile; 3 Center of Interdisciplinary Biomedical and Engineering Research for Health (MEDING), Universidad de Valparaíso, Valparaiso, Chile; 4 Faculty of Science, Department of Mathematics, Pontificia Universidad Javeriana, Bogotá, Colombia; 5 Biomedical Engineering School, Universidad de Valparaíso, Valparaiso, Chile; 6 Translational Research Center in Neuropharmacology (CItNE), Universidad de Valparaíso, Valparaiso, Chile; 7 Medical School, Universidad de Valparaíso, Valparaiso, Chile; 8 Millennium Institute for Intelligent Healthcare Engineering (iHealth), Santiago, Chile

**Keywords:** Epidemiological modeling, Spatiotemporal modeling, Deep learning, Dengue risk mapping, Climate sciences, Diseases, Ecology, Ecology, Environmental sciences, Environmental social sciences, Mathematics and computing

## Abstract

Understanding dengue dynamics from a spatiotemporal perspective has become increasingly relevant in recent years, as it allows the identification of factors influencing disease transmission, including climatic conditions, human behavior, and the distribution of *Aedes aegypti*, the primary vector responsible for dengue virus transmission in tropical and subtropical regions. This knowledge is essential for supporting public health decision-making and reducing the impact of dengue on vulnerable populations. This study proposes a spatio-temporal deep learning framework based on the U-Net++ architecture to generate high-resolution dengue risk maps in Colombia. The model integrates climatic, environmental, demographic, and socioeconomic information derived from satellite and census sources. Two spatial approaches were evaluated: a high-dimensional geography (*HDG*) approach at the national scale and a low-dimensional geography (*LDG*) approach applied to five departments with high dengue incidence. The model integrating climatic, social, and environmental information achieved the best performance in both approaches. In the *HDG* configuration, the best model (M9) reached a test mIoU of 0.6646 (validation mIoU = 0.7361). In the *LDG* configuration, the corresponding model achieved an average test mIoU of approximately 0.73 across departments. These results highlight the potential of integrating heterogeneous climatic, environmental, and socioeconomic data within a spatiotemporal deep learning framework to characterize dengue risk patterns and support high-resolution surveillance.

## Introduction

Dengue is the most common arboviral disease worldwide^1^. The World Health Organization *(WHO)* estimated that, by 2024, nearly 50% of the world’s population was at risk of dengue infection, with between 100 and 400 million cases reported annually^2^. The dengue virus *(DENV)* is transmitted to humans through the bite of *Aedes aegypti*, the primary vector of the disease, which is present in 167 countries worldwide. Although its distribution is practically global, its highest prevalence is concentrated in tropical and subtropical countries^3^.

Dengue represents a growing threat to public health on a global scale. In Colombia, the reported dengue incidence reached 251.88 cases per 100,000 inhabitants in 2023, compared with 134.33 in 2022, reflecting a worrying epidemic dynamic. This increase forms part of a wider regional pattern in Latin America, where 12.6 million cases were reported in 2024, the largest epidemic ever recorded in the region^4^.

Several factors have contributed to the increase in the global burden of dengue, including climate change, population growth, deforestation, unplanned urbanization, and human mobility^5–8^. In particular, countries with a medium and medium-low Socio-demographic Index *(SDI)* bear a greater disease burden, which is associated with high population density, precarious living conditions, and accelerated urbanization^6^. Likewise, climatic variables such as temperature and precipitation directly influence the population dynamics of *Aedes aegypti* by affecting the biting rate and the extrinsic incubation period, as well as by regulating vector reproduction and survival, which are key factors in the spread and transmission of DENV^7–10^. Therefore, understanding the spatiotemporal distribution of dengue incidence and identifying incidence-based risk areas through approaches that integrate climatic, environmental, and demographic factors is essential to support more effective, targeted, and long-term prevention and control strategies.

This article is organized as follows. Related work section reviews the main background and related work. Materials and methods section describes the study region under both the high-dimensional geography (*HDG*) and low-dimensional geography (*LDG*) approaches, detailing the data sources, variable construction, and modeling framework. Results section presents the results, while Discussion section discusses the main findings, conclusions, and future research directions.

## Related work

Over the last decade, access to open data, satellite remote sensing, and deep learning techniques has transformed approaches to dengue incidence modeling and incidence-based risk mapping. The literature can be grouped into two main areas: (i) statistical and Bayesian models representing spatiotemporal dependence in dengue incidence, and (ii) deep neural network architectures applied to multi-time series and remote sensing maps for incidence prediction or outbreak forecasting.

Dengue epidemic modeling frequently relies on linear regression, random forest or support vector machines for spatio-temporal analysis^11^. By incorporating socioeconomic, demographic, climatic, and environmental data, these techniques map disease risk in endemic areas and identify risk factors and dengue incidence. Nevertheless, data quality is a critical limitation for these machine learning algorithms, as their predictive performance depends directly on it^11^.

Epidemic models must guide the implementation of effective disease prevention and control measures at the most opportune times and locations. However, previous techniques show low accuracy in predicting the spatiotemporal distribution of dengue, which limits the modeling of complex dynamics influenced by multiple factors^12^. To overcome these restrictions, deep learning techniques combined with satellite remote sensing data at smaller spatial scales have been explored to predict the distribution of dengue outbreaks with higher accuracy^12^.

Another more flexible hierarchical and temporal models have been explored. Zhu et al.^13^ used a geographically and temporally weighted regression *(GTWR)* model in Yunnan, China (2013–2019) identifying positive effects of temperature, relative humidity, and imported cases, and adverse effects of precipitation and wind. Complementarily, Galeana et al.^14^ applied a Bayesian *BYM2* model to assess the role of deforestation and climate in Mexico (2010–2020), comparing three temporal structures: first-order autoregressive *(AR1)*, first-order random walk *(RW1)*, and second-order random walk *(RW2)*. They results showed that smoothed temporal structures *(RW2)* and spatial correlation improved model fit and epidemiological inference.

Other authors have incorporated spatial and fractal analysis techniques. Fernandes et al.^15^ combined the Moran index with multifractal analysis *(MFDFA)* to explore the temporal persistence of dengue outbreaks in Brazil, highlighting the combined role of climate and human mobility. These works demonstrate methodological advances, although they still rely on coarse spatial resolutions and, in some cases, restrictive assumptions regarding the functional form of the relationships between covariates and dengue incidence.

On the other hand, deep learning models offer a promising framework for representing nonlinear relationships and long-range temporal dependencies present in the spread of disease transmited of mosquitoes like dengue and malaria. Recent literature applying these techniques can be explicitly distinguished into three main predictive tasks: incidence forecasting (predicting case counts), outbreak detection, and spatial risk mapping (producing spatial risk surfaces).

In the context of incidence forecasting, Pillay et al.^16^ developed a Transformer model to predict malaria cases in South Africa (1998–2021) integrating climate variables and teleconnections, including El Niño, the Indian Ocean Dipole *IOD*, and the Southern Annular Mode *SAM*. Their model outperformed XGBoost and the distributed Southern lag nonlinear models *DLNMs*, demonstrating deep learning’s ability to retain temporal memory without relying on fixed lag structures, albeit with lower causal interpretability. This malaria study is relevant to the spatiotemporal modeling of dengue, as both vector-borne diseases coexist in tropical regions and share similar transmission dynamics. Therefore, understanding the complex relationships between different factors and malaria spread provides valuable methodological insights applicable to dengue.

Regarding dengue, Nguyen et al.^17^ applied four deep learning models for two predictive tasks in Vietnam: three-month-ahead incidence forecasting and outbreak detection for large-scale epidemics. The models analyzed included a Convolutional Neural Network *(CNN)*, a Transformer model, and attention-enhanced long short-term memory models *(LSTM-ATT)*. The models were trained (1997–2013) and tested (2014–2016) using dengue incidence rates and twelve meteorological variables–including temperature, humidity, precipitation, evaporation, and sunshine duration–recorded across 20 provinces between 1997 and 2016. The authors found that model performance improved when a three-month retrospective window was applied. Each model was evaluated independently for every province, and the results indicated that *LSTM-ATT* consistently outperformed the other models in predicting dengue incidence. Although the authors used a temporal data split to simulate future predictions, they acknowledge it lacks true temporal holdout validation. Consequently, this retrospective approach may overestimate the model’s real-world performance.

In the context of incidence forecasting, Anno et al.^12^ implemented a U-Net-type convolutional network to forecast outbreaks in Taiwan, integrating satellite epidemiological and climate data. The model improved the spatial and temporal representation of outbreak occurrence, but did not incorporate demographic or socioeconomic factors, which are key to characterizing territorial vulnerability and incidence-based risk.

The cited deep learning frameworks vary considerably in their spatiotemporal resolutions. Temporally, data aggregation ranges from daily records over 23 years for malaria prediction^16^, to biweekly intervals to mitigate zero-inflated daily counts^12^, and monthly steps for long-term dengue forecasting^17^. Spatially, analysis scales from broad provincial levels–either focused on a single region^16^ or independently across 20 distinct provinces^17^ –to a highly granular 1 km^2^ grid mapping (*384 × 512* pixel matrices) across Taiwan^12^. Consequently, direct comparisons of their predictive accuracies must be interpreted with caution. The performance and overall applicability of these epidemiological models depend heavily on data availability and temporal coverage, as longer or more granular datasets may better capture complex spatiotemporal dependencies.

To overcome previous limitations this study proposes a modeling framework based on the U!-Net++ architecture to characterize spatiotemporal patterns of dengue incidence and identify incidence-based risk categories in Colombia between 2007 and 2019. Unlike previous work, to the best of our knowledge, this approach integrates climatic, environmental, demographic, and socioeconomic data from satellite and census sources across two geographic scales: high-dimensional geography *(HDG)* at the national level and low-dimensional geography *(LDG)* at the level of five high-incidence departments. This framework contributes to the methodological development of high-resolution dengue-risk mapping and may provide complementary information for spatial epidemiological surveillance.

## Materials and methods

This section describes the methodological framework adopted to predict spatio-temporal patterns of dengue incidence and classify incidence-based risk categories in Colombia. First, the study area, dataset characteristics, and data sources are described. Next, the construction of the system variables is presented, including variables obtained from observed sources and derived variables based on interactions, moving windows, and anomaly measures. Finally, the experimental design is detailed, including the model training protocols and the metrics used to evaluate model performance.

### Study area

**Fig. 1:**
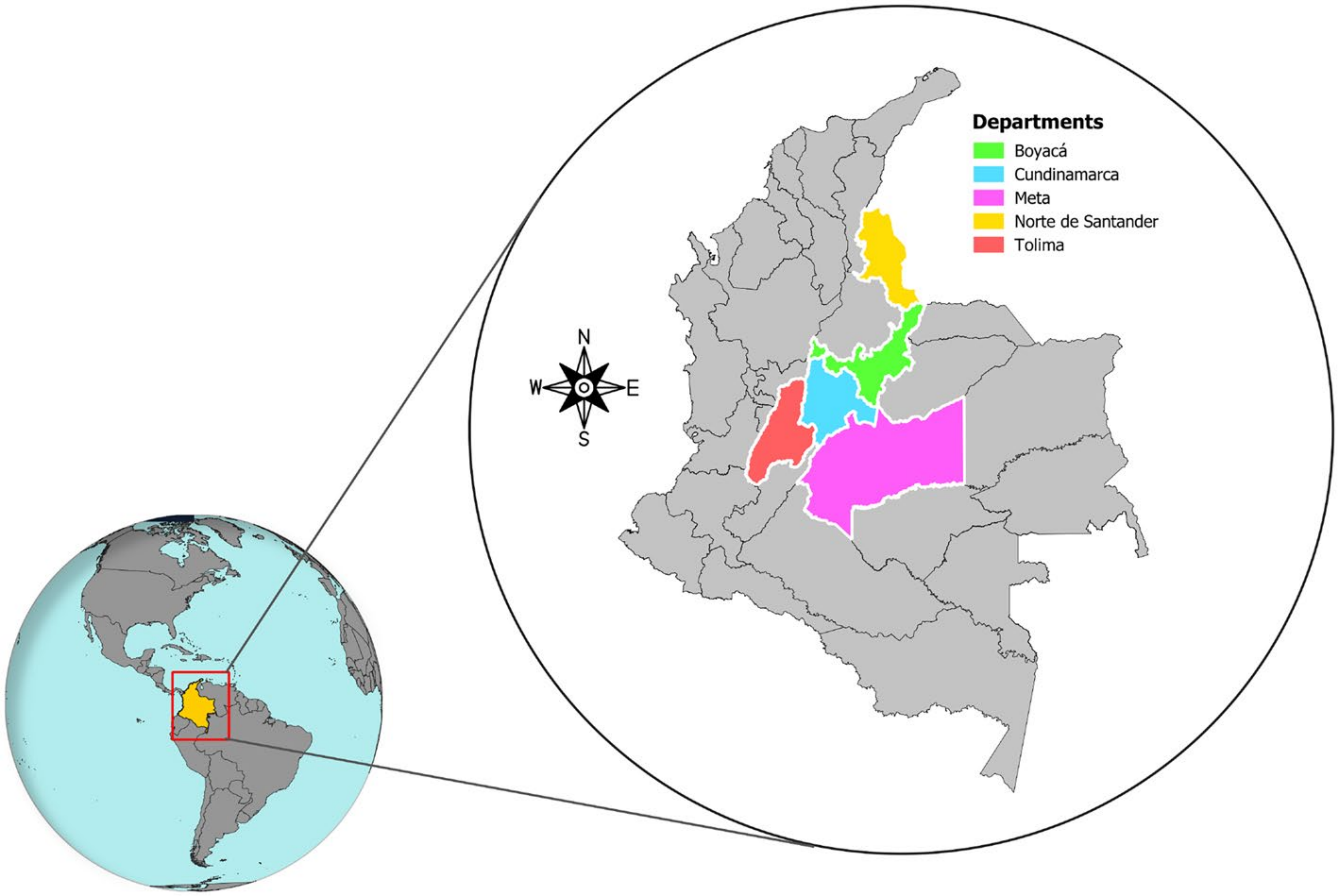
Map of Colombia with selected departments. Highlighted departments include Boyacá (123 municipalities), Meta (29 municipalities), Cundinamarca (116 municipalities), Tolima (47 municipalities), and Norte de Santander (40 municipalities), which were selected for spatiotemporal U-Net++ models. Maps were prepared using QGIS version 3.42 (https://qgis.org). The world vector basemap was provided by QGIS, and the administrative boundaries of Colombia were obtained from the Colombian Spatial Data Infrastructure (ICDE; https://www.icde.gov.co).

**Fig. 2:**
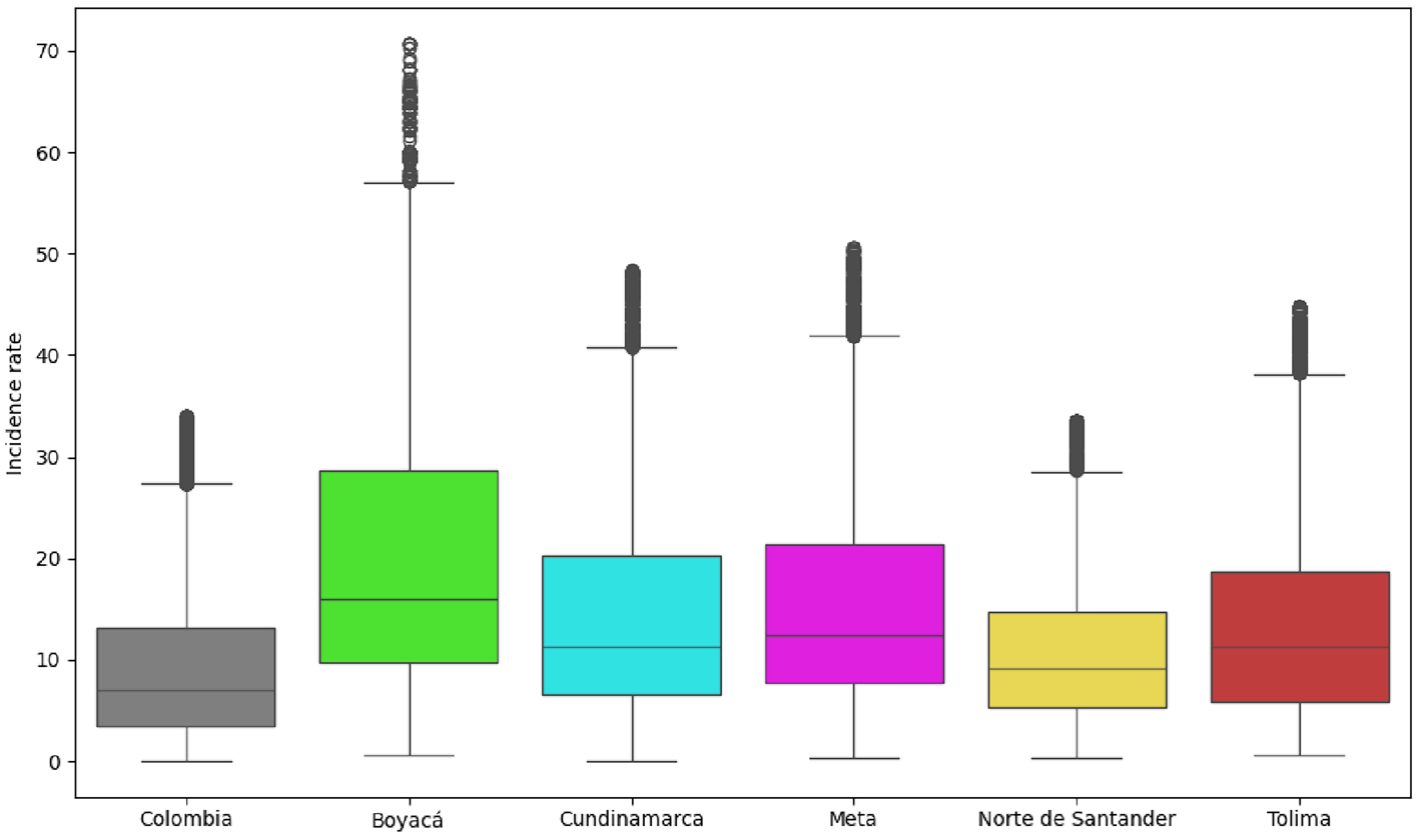
Box plot incidence rate for Colombia and selected departments (without extreme anomalies).

Colombia is located in South America, in the equatorial zone (See Fig. 1), and therefore does not experience the four traditional seasons observed in many parts of the world. Instead, its climate is characterized by alternating dry periods (December to January and July to August) and rainy seasons (April to May and October to November). The country is administratively divided into six regions (Caribbean, Andean, Orinoquia, Amazonian, Pacific, and Insular), comprising 32 departments and 1103 municipalities. Its marked topographic diversity gives rise to a wide range of landscapes, ecosystems, and climatic conditions. The study area comprised the entire continental Colombian territory under the high-dimensional geography (*HGD*) approach and five selected departments under the low-dimensional geography (*LGD*) approach. These departments were not excluded from the HDG analysis; rather, they were additionally modeled independently within the LDG framework to evaluate whether regional models could better capture local climatic, environmental, and socioeconomic heterogeneity. These selected departments exhibited first-quartile incidence rates higher than the national value (Q1 = 3.72) during the period analyzed: Boyacá (Q1 = 10.356), Meta (Q1 = 7.926), Cundinamarca (Q1 = 6.922), Tolima (Q1 = 6.058) and Norte de Santander (Q1 = 5.598) (see Fig. 2).

### Data sources and system variables

**Fig. 3:**
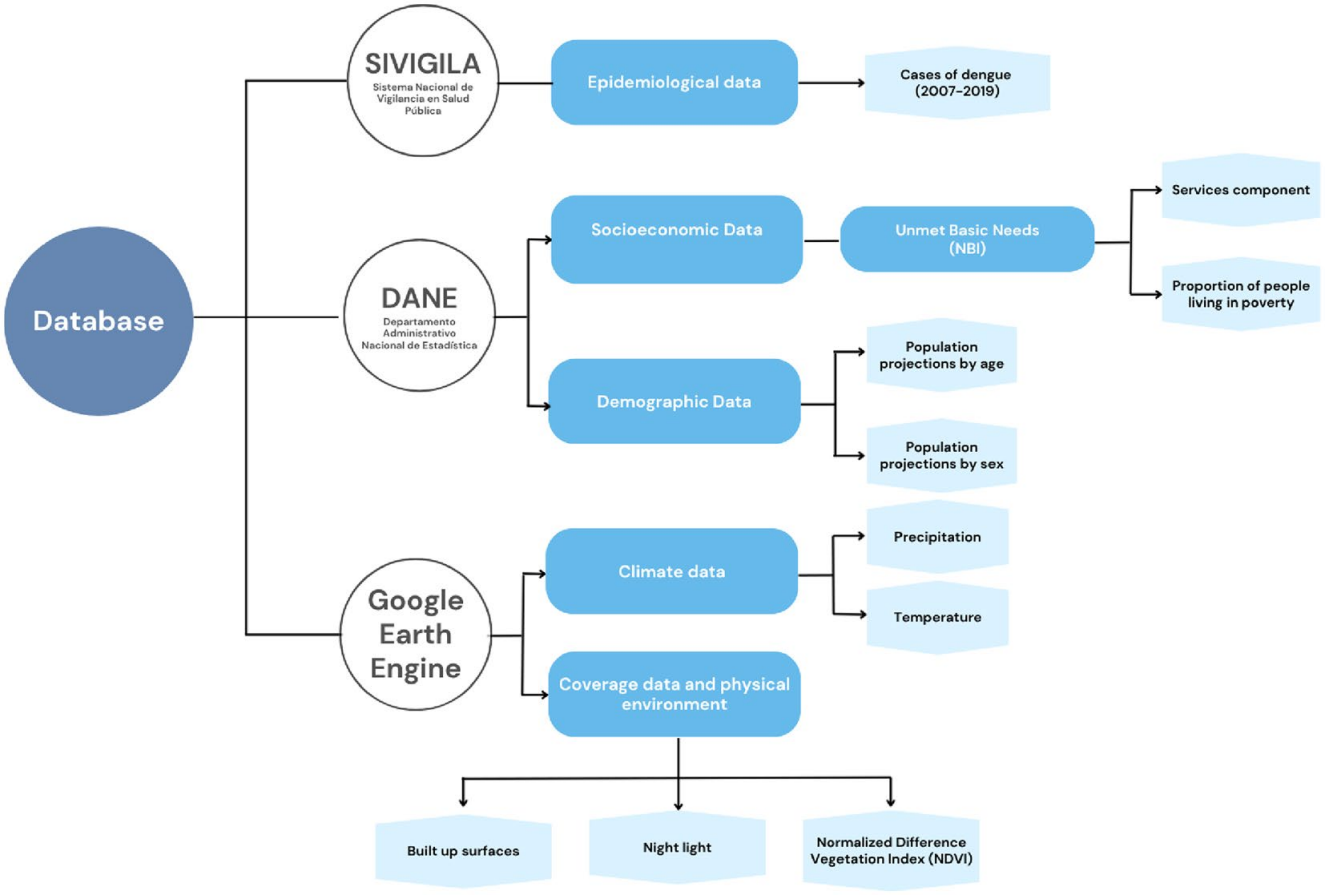
Sources and structure of the integrated database. Epidemiological data were retrieved from SIVIGILA; demographic and socioeconomic indicators from DANE (including NBI, PM, and SI); and climate and land-cover variables (temperature, precipitation, NDVI, built-up surfaces) were derived using Google Earth Engine.

We constructed a multi-source time-series dataset integrating short-term climate information, physical and biophysical territorial structure, human activity patterns, persistent social context, and lagged epidemiological information. Derived layers, including anomalies, moving windows, and interaction terms, were also incorporated to capture nonlinear relationships between factors. Data preparation included preprocessing and normalization adjusted to each source’s statistical distribution, spatial resolution, and temporal structure. The resulting model inputs were organized as stacks of spatial layers. The observed variables are summarized in Fig. 3.

The epidemiological information (2007–2019) was obtained from the National Public Health Surveillance System *(SIVIGILA)*^18^. The response variable was initially expressed as dengue incidence rates per 100,000 inhabitants, calculated from weekly cases, according to epidemiological weeks *(EW)*, and the annual municipal population. In addition, lagged incidence maps were incorporated as epidemiological memory. These maps were encoded in one-hot (0, 1) format and weighted by a decreasing function of temporal distance, allowing the model to preserve temporal coherence without replicating the most recent observed incidence pattern.

Sociodemographic and socioeconomic data were obtained from DANE^19^, including population projections by sex *(PPS)* and age *(PPA)* updated from the National Population and Housing Census CNPV^20^. The sex-specific layers were included to represent potential differences in exposure, mobility, and access to healthcare, while the age distribution layers, grouped into 0–4, 5–14, 15–49, 50–69, 70+ years, were used to represent heterogeneity in susceptibility and contact patterns. Finally, the Unsatisfied Basic Needs *(NBI)* indicator was included to characterize structural vulnerability, considering the proportions of people experiencing poverty-like deprivation *(PM)* and inadequate services *(SI)* for 2005 and 2018.

The satellite data sources and availability periods are detailed in Table 1. These layers form the basis of the environmental component of the modeling system, integrating climate and land-cover observations obtained from different optical and microwave sensors. All products were accessed and processed using the Google Earth Engine (GEE) platform^21^. Derived layers, such as anomalies, moving averages, and extreme event indicators, were calculated from the listed datasets.

**Fig. 4:**
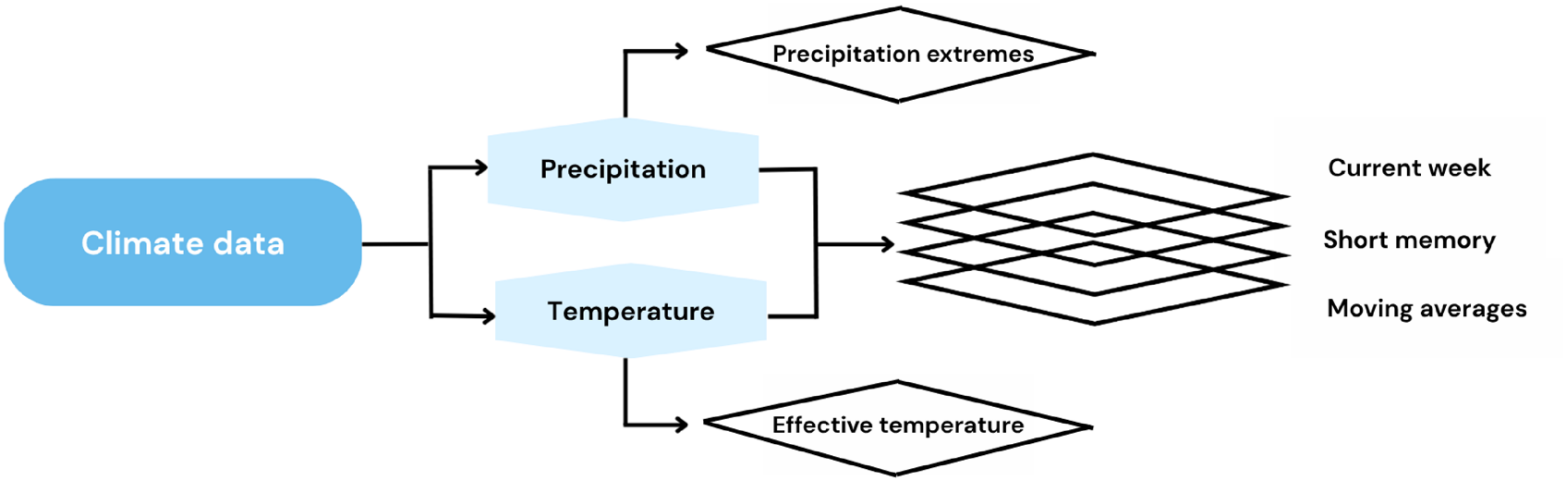
Schematic representation of the climatic variables used by the system. The climate data are decomposed into precipitation and temperature components, from which variables such as precipitation extremes, effective temperature, and temporal aggregations (current week, short-memory, and moving averages) are computed.

Figure 4 shows the integration of weekly climate information on precipitation (*P*) and land surface temperature (*T*). The rescaled weekly precipitation was used to represent short-term hydrological forcing, while land surface temperature was included because of its role in modulating vector and virus development and survival rates. To capture the biological time lag of climate effects on dengue incidence, lags from t*-1* to t*-4* and lagged moving averages, such as t*-2* to t*-6*, were incorporated. In addition, indicators of extremely wet events and derived thermal variables, such as *degree-days* and the proportion of observations above the thermal threshold, were added to approximate sustained environmental conditions conducive to dengue transmission. Finally, to represent interactions potentially associated with increased dengue incidence, dynamic versions of the demographic and socioeconomic layers were generated by multiplying them by recent climate forcing or nighttime light anomalies. These derived layers capture how social context and environmental conditions may jointly contribute to incidence-based risk categories.

**Table 1:** Satellite data sources used in the study and their exact identifiers in Google Earth Engine (GEE).

Variable	Sensor / Satellite	Product	Exact ID in GEE	Period
*T* (Land Surface Temperature)	MODIS Terra / Aqua	MOD11A1 (Terra) / MYD11A1 (Aqua), Collection 6.1	MODIS/061/MOD11A1, MODIS/061/MYD11A1	2007–2019
*P* (Precipitation)	CHIRPS (UCSB–CHG)	CHIRPS Daily Precipitation	UCSB-CHG/CHIRPS/DAILY	2007–2019
*NDVI* (Vegetation Index)	AVHRR (NOAA) / VIIRS (NOAA)	NOAA Climate Data Record (CDR) NDVI	NOAA/CDR/AVHRR/NDVI/V5 (2007–2013); NOAA/CDR/VIIRS/NDVI/V1 (2014–2019)	2007–2019
*BS* (Built-up Surface)	Landsat TM / ETM+ / OLI	GAIA v10 (FROM-GLC)	Tsinghua/FROM-GLC/GAIA/v10	2007–2019$\vphantom{0}^*$
*NTL* – Stage 1	DMSP/OLS	Harmonized Nighttime Lights (Harmonized NTL)	projects/sat-io/open-datasets/Harmonized_NTL/dmsp	2007–2013
*NTL* – Stage 2	VIIRS-DNB (NOAA)	Monthly VCMSLCFG Composites v1	NOAA/VIIRS/DNB/MONTHLY_V1/VCMSLCFG	2014–2019
*NTL* – Backup	VIIRS-DNB (NOAA)	Annual Composites v2.1	NOAA/VIIRS/DNB/ANNUAL_V21	2014–2019

* GAIA v10 was available until 2018; it was replicated from 2019 due to year-over-year stability in urban coverage

Land cover and physical environment information included built-up areas *(BS)*, nighttime radiance *(NTL)*, and the normalized difference vegetation index *(NDVI)*. The percentage of urban or built-up land, rescaled to [0, 1], was used as a proxy for urban intensity and for the physical habitat structure in which human-vector contact may occur. This layer represents built-up areas on an annual scale. The *NDVI*, normalized weekly to the range [0, 1], was used to characterize vegetation cover, with lower values tipically observed in dense urban areas and higher values in periurban or rural areas. To identify vegetation disturbances associated with drought or land-cover changes, the *NDVI* anomaly was calculated as the difference between a recent average and a longer temporal reference. Based on the combination of urbanization, for example, a high proportion of built-up areas and low vegetation, and the lack of basic services, an urban habitat index was constructed to synthesize, in a single layer, the degree of structural favorability for vector establishment in human environments.

Night-time light *(NTL)*, derived from the VIIRS sensor, was used as an indicator of human activity and infrastructure density, after rescaling the radiance to the range [0, 1]. This indicator was considered a proxy for mobility, occupancy, and space use. From this layer, the night-time light anomaly was calculated as the ratio of a recent average to a longer baseline, allowing the detection of disturbances, such as blackouts, events, or changes in activity patterns, that are not calculated by static layers. Likewise, a dasymetric redistribution based on night-time radiance was applied to reweight aggregated municipal layers, such as population or *(NBI)*, concentrating them on effectively occupied and illuminated areas and avoiding the assumption of territorial homogeneity. Finally, a municipal calibration of night-time radiance was carried out using *(NBI)* as a socioeconomic proxy, improving comparability between territorial units.

Interactions between environmental factors and habitat components were represented through products of climatic variables, nighttime radiance, and the urban habitat index. Rather than establishing new epidemiological mechanisms, these interaction terms were designed to encode relationships previously associated with dengue transmission in the literature. For example, rainfall and urban environmental conditions have been linked to the availability of *Aedes aegypti* breeding habitats, while nighttime radiance has been widely adopted as an indirect indicator of human activity and population concentration. Explicit seasonality was incorporated through sine and cosine transformations of the epidemiological week, enabling the model to capture recurrent temporal patterns related to climatic variability and vector dynamics without imposing a rigid temporal structure.

### Experimental and modeling framework

**Fig. 5:**
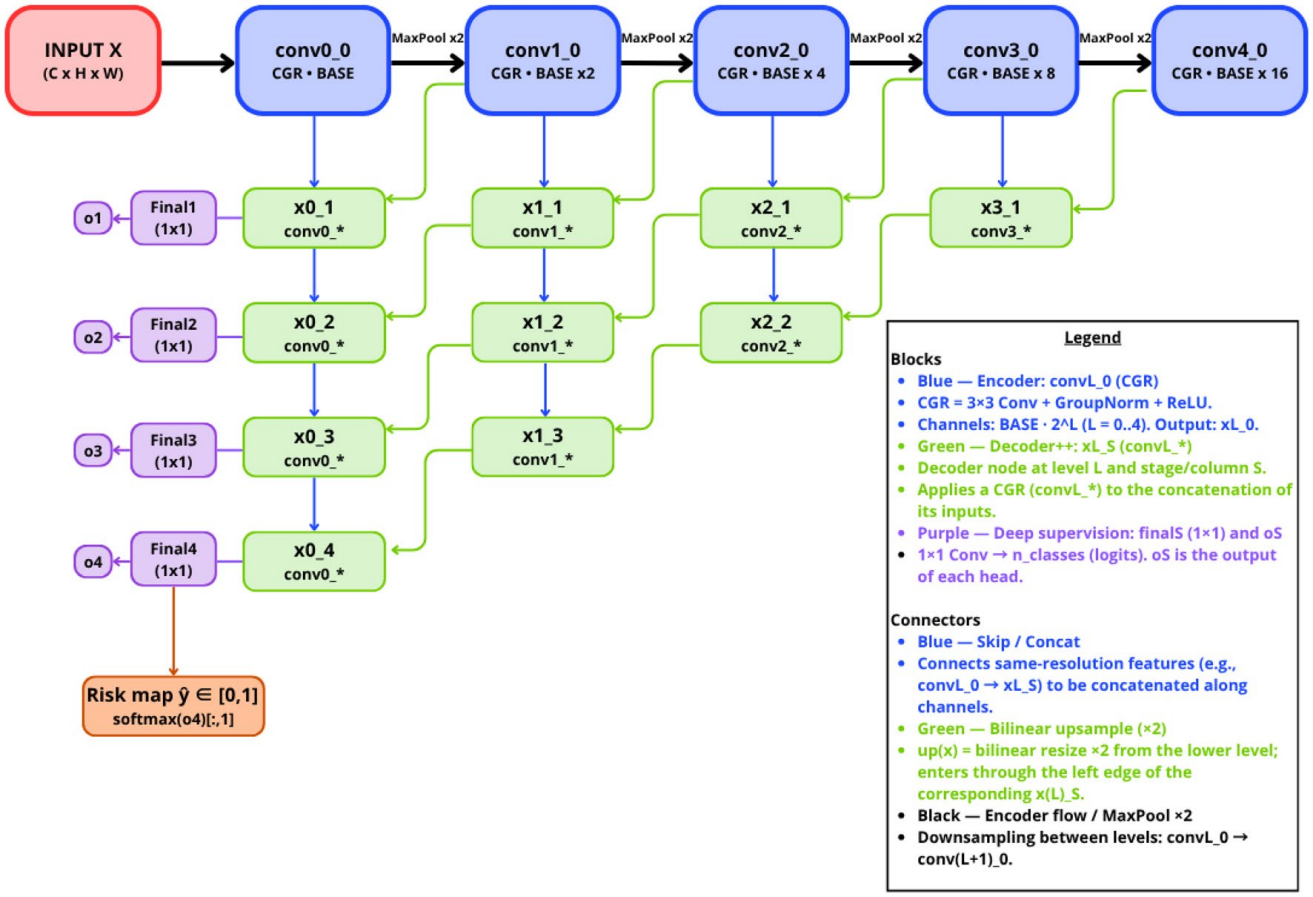
General workflow of the U-Net++ model for predicting spatiotemporal dengue incidence patterns and classifying incidence-based risk categories.

The chosen architecture is U-Net++, introduced by Zhou et al.^22^, an evolution of classic U-Net^23^, that introduces a mesh of dense connections between encoder and decoder. Instead of a single shortcut per level, U-Net++ creates a *family* of intermediate paths that refine features at multiple resolutions before each fusion in the decoder. On maps with acceptable, irregular boundaries between 0 and 1, the decoder must not rely on a single long path to reconstruct contours. Instead, it receives pre-sharpened versions of the same information at different scales, reducing sawtooth artifacts and eliminating vague areas at boundaries. This architecture was selected for its proven ability to capture non-linear relationships and spatial hierarchies in geospatial raster data, outperforming conventional CNNs and standard U-Net models in segmentation and prediction tasks. Figure 5 shows a U-Net++ with five levels: the *encoder* (blue) applies CGR blocks and downsamples with $\text {MaxPool}\times 2$; the *decoder* (green) reconstructs with bilinear $\text {upsampling}$ and dense connections $x_{i,j}$ within each column. Four deep supervision heads (Final 1–Final 4) are added to the nodes $x_{0,j}$, each with $\text {Conv }1\times 1 \rightarrow 2$ *logits* $(o_j)$. During training, a weighted sum $\sum _{j=1}^{4} w_j\,\mathcal {L}(o_j, y)$ is minimized. The model’s final output is obtained from the last deep supervision head (Final 4). The *logits* $(o_{4}$ are transformed by a *softmax* function, and the probability corresponding to the positive (risk) class is retained as the model output: $\hat{y} = \big [\textrm{softmax}(o_{4})\big ]_{1}$. Thus, $\hat{y}$ represents the continuous spatial probability map of risk.

The model was implemented in the PyTorch environment with GPU acceleration. Each input patch of 128*×*128 pixels was processed through a five-level encoder-decoder structure (four reduction stages), with convolutional blocks composed of Conv(3*×*3) operations, group normalization (GroupNorm), and ReLU activation. The number of filters was doubled at each level, starting from a base of 16 channels. Dropout was applied to the prior incidence channels to reduce overfitting. Training was performed using the AdamW optimizer, with gradient accumulation equal to 8 and a learning rate defined in the training script. The loss function chosen was cross-entropy (2 classes) with class weights, ignoring pixels outside the valid area, incorporating deep supervision, and a label smoothing factor of 0.05. Training was performed for up to 100 epochs with early stopping (patience = 10) (see Table 2).

Two geographic modeling configurations were evaluated: (i) High-dimensional geography *HDG*, corresponding to a national model trained using data from municipalities in Colombia, and (ii) Low-dimensional geography *LDG*, consisting of independent models fitted separately for each of the five selected departments exhibiting high epidemiological variability. To assess model performance under different levels of data availability and recency, three temporal horizons were considered: (a) 2007–2019 (full historical series), (b) 2016–2019 (recent medium-term period), and (c) 2018–2019 (short-term period). To avoid information leakage and preserve the temporal structure of dengue dynamics, the data were partitioned using a strictly chronological split. For each temporal horizon, the earliest observations were assigned to the training set, the subsequent observations to the validation set, and the most recent observations to the test set, following a 70/15/15 ratio. This partitioning was applied consistently in both the *HDG* and *LDG* configurations. Municipalities included in the training set could also appear in the validation and test sets; however, only observations from later time periods were used. Consequently, no information from future weeks was available during model training, ensuring temporal independence between the training, validation, and test subsets. In the *LDG* configuration, each departmental model was trained and evaluated exclusively using municipalities belonging to the same department, whereas the *HDG* model was trained and evaluated using municipalities from across Colombia. Accordingly, the proposed evaluation framework assesses the models’ ability to generalize to future temporal conditions not observed during training while maintaining the underlying spatial structure of the study area. The results should therefore be interpreted as evidence of temporal transferability rather than spatial extrapolation to previously unseen municipalities.

**Table 2:** Main hyperparameters of the U-Net++ model.

Parameter	Value	Description
Input patch size	*128 × 128*	Training crop size.
Number of levels	5 (4 downsamples)	Encoder depth: conv0_0 ... conv4_0.
Filters per layer	base *=16*; [16, 32, 64, 128, 256]	Channels double each level (base_ch = 16).
Activation	ReLU	CGR blocks: Conv($3{\times }3$) + GroupNorm + ReLU.
Optimizer	AdamW	Gradient accumulation = 8; LR set in training script.
Loss function	Cross-Entropy (2 clases)	Deep supervision: sum over $o_1..o_4$; label smoothing 0.05.
Epochs	100 (early stop)	Early stopping patience *=10*.

Incidence rates were initially classified into four categories –**0**:0–1 cases per 100, 000 inhabitants (Zero or near-zero incidence), **1**:1–10 (Low), **2**:10–20 (Medium), and **3**: greater than 20 (High)–, thereby defining a multiclass classification problem. However, the dataset exhibited a *severe class imbalance*, which hindered stable training and accurate recovery of minority classes. In the 2018–2019 training set (72 files; 24,250,176 valid pixels), class 0 accounted for 81.8% of the pixels, whereas classes 1–3 represented only *18.2%* (*6.31%*, 5.46%, and *6.43%*, respectively). In the validation set (15 files; 5,052,120 pixels), class 0 represented for *63.16%* of the pixels, while classes 1–3 accounted for *6.48%*, *11.32%*, and *19.05%*, respectively. Similarly, in the test set (17 files; 5,725,736 pixels), class 0 represented *69.90%*, compared with $8.92\%, 9.91\%\,\,\text {and}\,\,11.27\%$ for classes 1-3. This imbalance became more pronounced when longer temporal windows were considered, leading to unstable decision boundaries and poor representation of minority classes.

To address this issue, the problem was reformulated as a binary classification task by grouping the classes $\{1,2,3\}$ into a single positive superclass:

$$y_{\text {bin}} \;=\; {\left\{ \begin{array}{ll} 0, & \text {si } y \in \{0\} \\ 1, & \text {si } y \in \{1,2,3\}. \end{array}\right. }$$

This decision was motivated by three considerations: (i) improved robustness to class imbalance through an increased number of positive samples; (ii) greater spatial stability by reducing confusion among adjacent incidence categories; and (iii) practical relevance, since the primary objective was to identify areas with incidence levels above the zero or near-zero reference category. Consequently, the final model outputs should be interpreted as binary incidence-based maps indicating the probability that a spatial unit belongs to the positive incidence class, rather than as estimates of dengue incidence intensity. All performance metrics reported in this study, including mIoU, correspond exclusively to this binary formulation. Therefore, the resulting predictions do not differentiate between low-, medium-, and high-incidence conditions, but rather distinguish between zero or near-zero incidence and the presence of an incidence-based positive class.

**Table 3:** Experimental configurations evaluated in the study.

Code	Model name	Description of included variables	No. variables
**M1**	Basic Climate	Temperature *T* and precipitation *P* (Current) + weekly incidence lags.	10
**M2**	Climate and epidemiological burden by sex	**M1** + Population projections by sex (M/F) (*PPS*).	12
**M3**	Climate–Demographic	**M2** + Population projections by age (5 intervals) (*PPA*).	17
**M4**	Climate, demographic and poverty	**M3** + proportion of people in situations of poverty (*PM*).	18
**M5**	Climate and Sociodemographic	**M4** + inadequate services (*SI*).	19
**M6**	Climate-Social-Environmental	**M5** + (*NDVI*).	20
**M7**	Climate-Social-Environmental and built-up	**M6** + Built up surfaces (*BS*).	21
**M8**	Climate-Social-Environmental complete$\vphantom{0}^{-}$	**M7** + Night light (*NTL*) (Without transformation).	22
**M9**	Climate-Social-Environmental complete$\vphantom{0}^{+}$	Same variables as **M8**, with transformations of *NTL*.	22
**M10**	Temporal Extended (PT-stacks)	8 (stacks *P*/*T*: Taking the current week of incidence to predict, *T* and *P* are used with lag $t-1\,\, to\,\ t-4$) + 12 (*PPS*, *PPA*, *PM*, *SI*, *NDVI*, *BS*, *NTL*) + 8	30
**M11**	Extended Exogenous (multi-source)	Extended configuration with 51 exogenous variables (8 stacks *P*/*T* + 9 weather in windows (*P*,*T*, extremes, quantile and NDVI window/anomaly) + 2 seasonality + 7 static (5 *PPA* and 2 *PPS*) + 14 dynamic ((5 *PPA* and 2 *PPS*) *×* (*NTL*,*P*))+ 2 SI dinamic + 2 PM dinamic + 1 hábitat H + 1 NDVI current + 1 NTL + 1 NTL anomalous + 1 BS + 2 NBI static) + 8 lags.	61

In all configurations, the eight channels corresponding to the weekly prior incidence lags remained active. Exogenous variables were obtained from satellite and census sources, harmonized at a uniform spatial resolution

Each model combines climatic, sociodemographic, and environmental variables, keeping the weekly lags of prior incidence active (4 lags *×* two classes = 8 channels).

Finally, multiple experimental configurations were designed to evaluate the influence of different determinants on the model’s predictive capacity, combining climatic, demographic, socioeconomic, and land-cover variables. Each configuration maintains the prior incidence (four weeks and two classes), ensuring temporal consistency of the inputs. Table  3 details the composition of each scenario and the total number of variables used, within a spatiotemporal predictive modeling framework.

### Evaluation metrics

Model performance was evaluated using the mean Intersection over Union (mIoU), a widely used metric in spatial segmentation and remote sensing tasks^24,25^. In this study, mIoU quantifies the spatial overlap between predicted and observed incidence-based classes, while simultaneously penalizing omission and commission errors. For a class *c*, the IoU is defined as

1 $$\begin{aligned} IoUc=\frac{TP_{c}}{TP_{c}+FP_{c}+FN_{c}}, \end{aligned}$$

where $TP_{c},FP_{c},FN_{c}$ correspond true positives, false positives, and false negatives, respectively. The mIoU is obtained by averaging the IoU values across all classes.

The mIoU was selected as the primary evaluation metric because the study’s objective was not only to assign each spatial unit to the correct incidence-based class, but also to assess the agreement between observed and predicted spatial patterns. Unlike overall accuracy, mIoU explicitly measures the spatial coincidence between predicted and observed class regions and is therefore particularly suitable for spatial segmentation tasks. In addition, mIoU is less dominated by the majority class than accuracy-based measures, making it more informative in imbalanced spatial classification tasks.

Although additional metrics such as precision, recall, F1-score, and AUROC are commonly used in classification problems, they were not considered primary evaluation criteria in this study because the main objective was the spatial delineation of incidence-based classes rather than pixed-wise classification performance alone. Therefore, the reported mIoU values should be interpreted as measures of spatial agreement between observed and predicted incidence-based categories, not as direct measures of incidence intensity or causal transmission risk.

During training, the validation mIoU, denoted as mIoU(val), was computed on the validation set to monitor the convergence and stability of the model U-Net++^26^. Subsequently, the test mIoU, denoted as mIoU(test), was estimated on the independent test set to assess generalization to data not used during model fitting or validation. In the context of dengue incidence-based spatial classification, similar values between validation and test sets indicate stable spatial learning and consistent predictive performance across the evaluated temporal and geographic partitions.

For each model configuration, the reported mIoU values correspond to the final performance obtained from a single training and evaluation run using the predefined training, validation, and test partitions. Consequently, no confidence intervals, repeated training runs, or formal statistical significance tests were computed. Therefore, comparisons among configurations should be interpreted as differences in observed performance rather than as evidence of statistically significant superiority. Throughout the manuscript, expressions such as higher observed performance” or best observed performance” are used to describe configurations achieving larger mIoU values.

## Results

The results obtained using the two proposed geographic approaches–HDG and LDG–are presented below for the three defined periods. Each configuration integrates different climatic, demographic, and environmental variables (See Table  3).

Since the original four-class incidence categorization was reformulated as a binary classification problem to address severe class imbalance, all results reported in this section correspond to the binary dengue risk prediction setting (presence versus absence of dengue risk). Consequently, all mIoU, IoU, and Dice values refer exclusively to this binary formulation.

This section summarizes the models’ performance and compares their predictive capacity, highlighting the most relevant spatiotemporal patterns identified at each analysis scale.

In the *HDG* approach, models were trained using the entire national territory to capture broad spatiotemporal patterns and evaluate temporal generalization at the national scale. The *Climate-Social-Environmental complete*$\vphantom{0}^{+}$ (**M9**) configuration exhibited progressively higher observed performance as the training window was restricted to more recent periods. Validation mIoU increased from 0.5122 in the 2007–2019 setting to 0.7361 in the 2018–2019 setting (*Δ* = +0.224), while test mIoU increased from 0.5331 to 0.6646 (*Δ* = +0.131) (Table 4). The larger improvement observed in validation relative to testing suggests that part of the gain may be associated with greater temporal proximity between training and evaluation data. This behavior may reflect reduced temporal variability, stronger temporal autocorrelation, or a greater contrast between dengue-risk and non-risk patterns in recent years. Because these factors were not evaluated separately, the observed performance gains should not be attributed to a single cause. Rather, they indicate that model performance is sensitive to the training window and may benefit from recent epidemiological conditions more similar to those encountered during evaluation. Since the 2018–2019 period yielded the highest observed validation and test mIoU values among the evaluated periods, it was selected as the reference period for subsequent comparisons across model configurations.

**Table 4:** Performance metrics of the U-Net++ model (Best model: **M9** Original Baseline: Climate-Social-Environmental complete$\vphantom{0}^{+}$) during validation by period and approach *HDG*.

Code	Period	mIoU_val_	mIoU_test_
**M9**	2007–2019	0.5122	0.5331
**M9**	2016–2019	0.6012	0.5856
**M9**	2018–2019	0.7361	0.6646

**Fig. 6:**
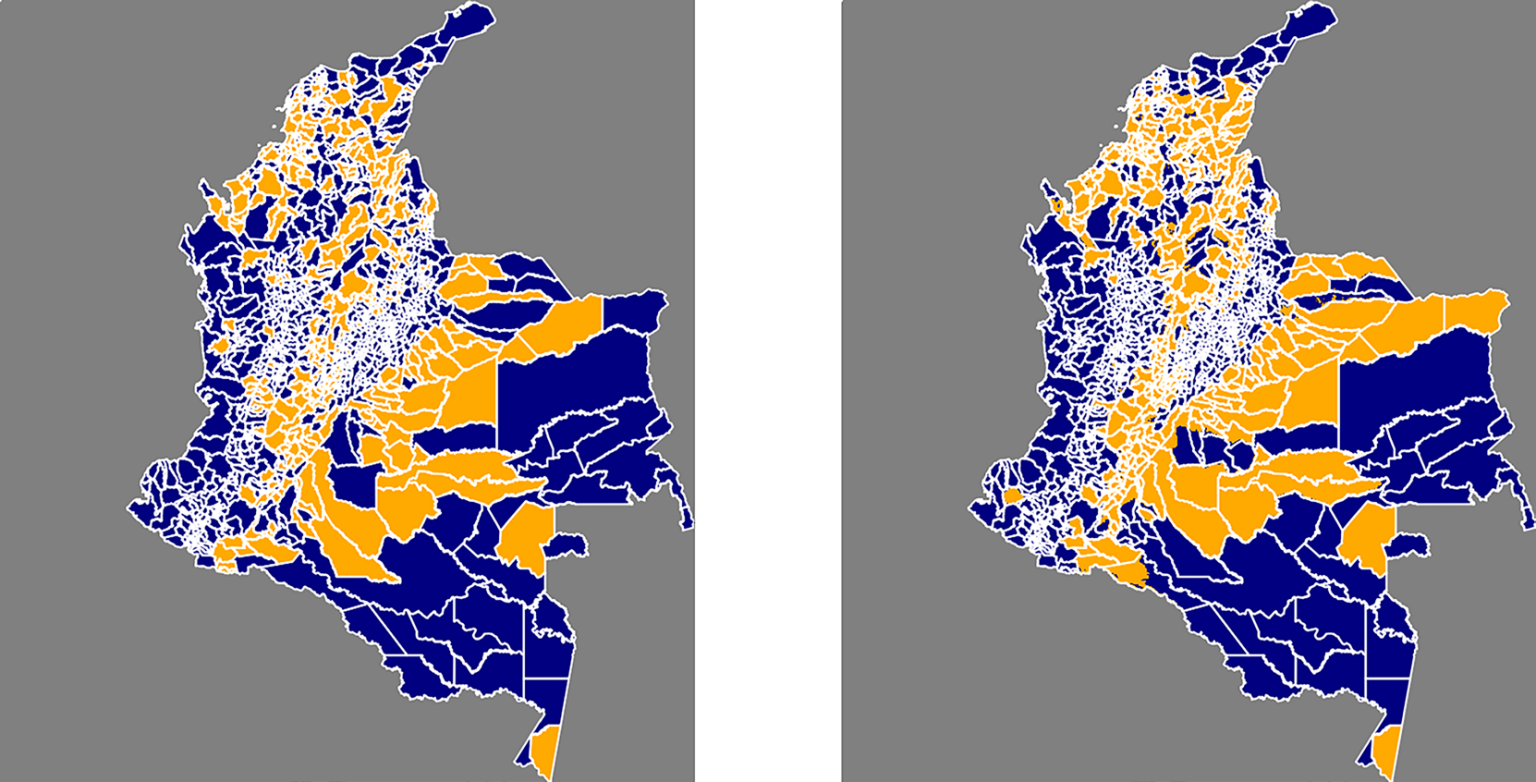
Experimental results for the test dataset in epidemiological week 36 of 2019. The left panel shows the observed binary incidence-based map, and the right panel shows the corresponding U-Net++ prediction using the combined climate–social–environmental data configuration. Blue pixels represent the zero or near-zero incidence class, whereas yellow pixels represent the positive incidence-based class. The maps were generated by the authors in Python using Matplotlib v3.8.4 (https://matplotlib.org/).

Table 5 summarizes the global validation and testing results for the 2018–2019 period. Models **M9**, **M10**, and **M11** correspond to the most robust phase of the experiment and represent different levels of complexity in integrating environmental and dynamic information. These three configurations achieved the highest observed validation and test mIoU values among the evaluated models, suggesting that the combined use of climatic, demographic, socioeconomic, and environmental variables may improve spatial discrimination of dengue risk. Figure 6 shows the dengue risk probability map generated by model **M9** for epidemiological week 36 of 2019 under the *HDG* approach. The predicted risk surface was broadly consistent with historically affected areas, particularly in regions characterized by elevated dengue incidence. While this agreement does not by itself demonstrate predictive skill, it provides qualitative support that the model captures relevant large-scale spatial patterns of dengue risk.

**Table 5:** Global results in validation and test with *HDG* approach (period 2018–2019).

Code	mIoU_val_	IoU_0 val_	IoU_1 val_	mIoU_0 test_	IoU_0 test_	IoU_1 test_
**M1**	0.6312	0.658	0.6042	0.5149	0.5648	0.4651
**M2**	0.6778	0.7134	0.6417	0.5645	0.6293	0.4997
**M3**	0.6750	0.7174	0.6326	0.5746	0.6492	0.5000
**M4**	0.6517	0.6823	0.6210	0.5260	0.5785	0.4734
**M5**	0.6639	0.6976	0.6300	0.5593	0.6190	0.4995
**M6**	0.7039	0.7468	0.6610	0.6206	0.6960	0.5451
**M7**	0.6866	0.7286	0.6445	0.5952	0.6682	0.5222
**M8**	0.6809	0.7205	0.6414	0.5638	0.6310	0.4965
**M9**	0.7361	0.7912	0.6809	0.6646	0.7609	0.5683
**M10**	0.7259	0.7834	0.6684	0.6116	0.7130	0.5102
**M11**	0.7143	0.7765	0.6521	0.6332	0.7325	0.5339

Under the *LDG* approach, the **M9** model also exhibited higher observed performance when trained on shorter and more recent temporal windows (see Table 6). Average validation mIoU increased from 0.4385 in the 2007–2019 period to 0.6988 in the 2018–2019 period, while test mIoU increased from 0.4815 to 0.6246. Similar to the HDG results, the larger gain observed in validation than in testing suggests that part of the improvement may be associated with the reduced temporal variability and greater homogeneity in the most recent periods. For the 2018–2019 setting, the model achieved a mean mIoU$\vphantom{0}_{\text {val}}$ of 0.6988, with moderate variability among departments (SD = 0.0385), and a mean test mIoU$\vphantom{0}_{\text {test}}$ of 0.6246. These results suggest that model performance may benefit from the use of more recent training data, although the smaller gains observed in the test set indicate that temporal non-stationarity remains a relevant challenge for generalization.

**Table 6:** Average performance metrics of the U-Net++ model (Best model: **M9** Original baseline: Full Socio-Environmental$\vphantom{0}^{+}$) during validation by period and approach *LDG*.

Code	Period	mIoU_val_	mIoU_test_
**M9**	2007–2019	0.4385	0.4815
**M9**	2016–2019	0.5810	0.5750
**M9**	2018–2019	0.6988	0.6246

Table 7 presents the results by department for the three configurations with the highest observed performance (**M9**–**M11**). Under the *LDG* approach, an independent model was trained and evaluated for each department. Therefore, the reported results assess temporal generalization within each region rather than spatial transferability across unseen departments. In contrast, the *HDG* approach evaluates a single national model trained on the entire Colombian territory and therefore assesses its ability to generalize across a broader range of spatial and temporal conditions. The mIoU_val_ values observed under LDG were generally high and exhibited relatively low variability across departments, suggesting a consistent level of temporal predictive performance across the evaluated regional settings. Model **M9** achieved the highest observed average validation performance (0.6988), with particularly high mIoU values in Boyacá (0.7390) and Cundinamarca (0.7110), both of which are characterized by substantial environmental heterogeneity.

**Fig. 7:**
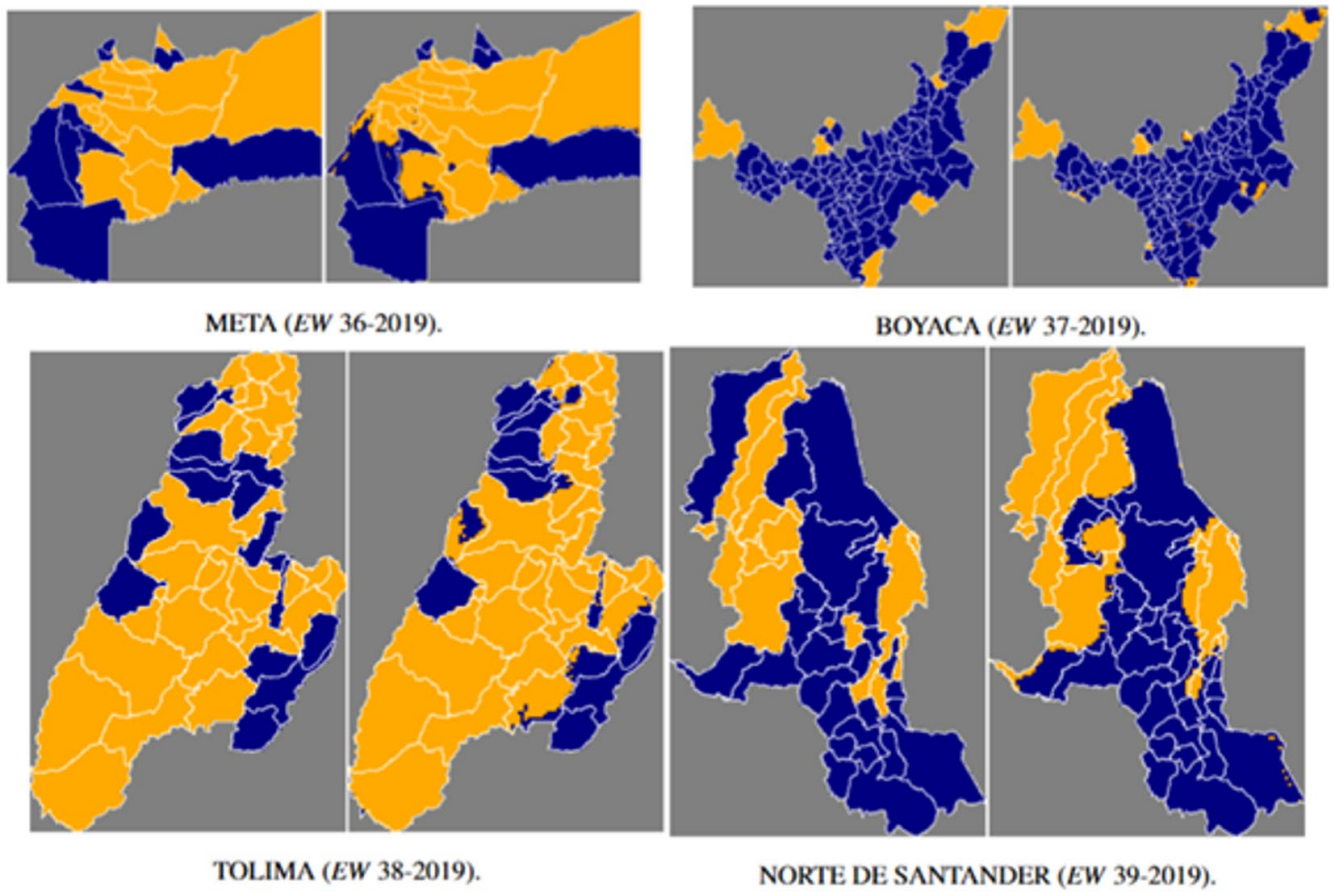
Experimental results for the departmental test datasets for selected epidemiological weeks of 2019. For each department, the left panel shows the observed binary incidence-based map, and the right panel shows the corresponding U-Net++ prediction using the climate–social–environmental complete+ data configuration. Blue pixels represent the zero or near-zero incidence class, whereas yellow pixels represent the positive incidence-based class. The maps were generated by the authors in Python using Matplotlib v3.8.4 (https://matplotlib.org/).

Although mIoU was used as the primary metric for model selection and comparison, class-specific IoU and Dice coefficients were also examined to provide additional insight into the model’s ability to identify dengue-risk areas. In particular, IoU_1_ and Dice_1_ quantify the agreement between observed and predicted pixels belonging to the positive dengue-risk class. These metrics were computed for all model configurations but are reported only for the best-performing models (**M9**–**M11**) to facilitate interpretation of class-specific predictive behavior. Consequently, they should be viewed as complementary indicators to mIoU rather than alternative model-selection criteria (Fig. 7).

**Table 7:** Global results in validation by department by **M9**, **M10** and **M11** (period 2018–2019).

Department	Code	mIoU_val_	IoU_0 val_	IoU_1 val_	Dice_0 val_	Dice_1 val_
BOYACA	**M9**	0.7390	0.9180	0.5610	0.9570	0.7190
	**M10**	0.6870	0.8910	0.4830	0.9420	0.6520
	**M11**	0.6330	0.8530	0.4130	0.9200	0.5840
CUNDINAMARCA	**M9**	0.7110	0.8020	0.6190	0.8900	0.7650
	**M10**	0.6890	0.7910	0.5870	0.8830	0.7400
	**M11**	0.7190	0.8360	0.6030	0.9110	0.7520
META	**M9**	0.7130	0.6290	0.7970	0.7710	0.8870
	**M10**	0.7153	0.6267	0.8039	0.7705	0.8913
	**M11**	0.7270	0.6502	0.8035	0.7881	0.8911
NORTE DE SANTANDER	**M9**	0.6360	0.6710	0.6010	0.8030	0.7500
	**M10**	0.6130	0.6270	0.5980	0.7710	0.7490
	**M11**	0.6180	0.6330	0.6030	0.7760	0.7520
TOLIMA	**M9**	0.6950	0.5640	0.8260	0.7210	0.9040
	**M10**	0.7070	0.5870	0.8280	0.7390	0.9060
	**M11**	0.7060	0.5880	0.8240	0.7410	0.9030

## Discussion

The results of this study illustrate the potential of a spatiotemporal deep learning framework to combine heterogeneous data sources and identify spatial patterns associated with dengue risk at high-resolution. In this study, *incidence* refers to the observed epidemiological rate used to construct the target variable, whereas *risk* refers to the binary spatial outcome derived from this incidence threshold and represented by the model as the presence or absence of dengue-risk areas. Therefore, the term *prediction* is used here to describe the model-based estimation of dengue-risk presence, rather than the direct prediction of incidence magnitude.

The strong performance observed in this study likely reflects the combined effect of three complementary components: (i) the representational capacity of the U-Net++ architecture to capture complex spatial patterns, (ii) the integration of climatic, environmental, socioeconomic, and demographic information, and (iii) the inclusion of prior dengue incidence, which provides epidemiological memory and temporal context. In particular, the *Climate–Social–Environmental complete*$\vphantom{0}^{+}$ model (**M9**) achieved the best performance at both the national (*HDG*) and departmental (*LDG*) levels. These results suggest stable temporal generalization within recent time windows under the evaluated binary dengue-risk mapping framework, although they should not be interpreted as direct evidence of incidence prediction or intensity estimation. The observed performance improvement as the analysis period becomes shorter indicates that predictive performance is partially influenced by temporal proximity and data homogeneity. Consequently, the model appears to generalize more effectively under recent epidemiological and environmental conditions, while performance tends to decrease when longer historical periods are included, reflecting potential sensitivity to temporal non-stationarity. Although the progressive performance gains observed when additional variables were incorporated support the relevance of socioeconomic and environmental information, the present experimental design does not allow the individual contribution of each component to be quantified. Therefore, the observed improvements should be interpreted as arising from the combined modeling framework rather than being attributed exclusively to the U-Net++ architecture, prior incidence information, or any specific group of predictors.

These findings are consistent with trends observed in recent studies highlighting the combined influence of climate, urbanization, and social vulnerability on dengue transmission^13–15^. This study, to our knowledge, is the first to employ a deep learning model to predict dengue risk in Colombia, explicitly considering the spatiotemporal behavior of the relationship between incidence and climatic, social, and environmental determinants. Unlike more conventional statistical approaches–such as hierarchical Bayesian models *(BYM2)* or spatiotemporal regression frameworks *(GTWR)*–the proposed model provides a highly flexible data-driven framework for learning complex spatial patterns and nonlinear relationships among climatic, environmental, and socioeconomic factors. While Bayesian and regression-based approaches can also account for spatial dependence and certain forms of nonlinearity, U!-Net++ is particularly well suited for extracting multiscale spatial features directly from gridded data through its encoder–decoder architecture and dense skip connections. This ability to preserve spatial coherence across multiple spatial resolutions may facilitate the representation of transitions between low- and high-risk areas and the characterization of complex spatial structures that are difficult to specify a priori in conventional modeling frameworks.

Incorporating socioeconomic and demographic variables was associated with improved model performance. Although the use of nighttime radiance as an indicator of human activity and population concentration is well established in the literature, its integration with environmental and climatic variables enabled the model to represent multiple dimensions of dengue risk simultaneously. The resulting probability maps for week 36 of 2019 showed strong spatial agreement with historically affected areas, providing empirical support for the epidemiological relevance of the detected risk patterns.

From an operational perspective, the proposed framework demonstrates the potential of deep learning for municipal-scale dengue-risk mapping and could serve as a foundation for future early warning systems. However, the present study was based on retrospective model evaluation and was not designed to assess real-time forecasting performance, operational latency, or specific prediction lead times. Therefore, the results should be interpreted as evidence of the model’s ability to identify spatiotemporal risk patterns under historical conditions rather than as a demonstrated operational early warning system. Nevertheless, the model’s performance across different geographic scales (*HDG* and *LDG*) suggests that the proposed framework could be adapted and further evaluated for integration into national surveillance programs and local public health decision-support systems.

However, the study has some limitations. The quality and periodicity of the epidemiological data may be affected by underreporting or delays in case reporting. Furthermore, differences in spatial resolution between satellite and census sources imply aggregation processes that could introduce uncertainty. In the case of GAIA, the absence of data beyond 2018 required extending the urbanization layer to 2019 under the assumption of relative interannual stability. Although this assumption is supported by the limited temporal variability observed in the dataset, it may not fully capture localized changes in urban expansion or land-use dynamics. Consequently, the results are partially dependent on the validity of this stability assumption, which could be challenged in contexts characterized by rapid environmental or socioeconomic change. An additional limitation arises from the binary formulation adopted for dengue-risk mapping. While this approach mitigated the severe class imbalance observed in the original multiclass problem, it does not allow the model to distinguish between low, medium, and high incidence levels. Consequently, the resulting risk maps indicate the presence or absence of dengue risk rather than differences in incidence intensity. Furthermore, the model does not explicitly quantify the uncertainty associated with its predictions, constituting an important avenue for future research.

A limitation of this study is that model comparisons were based on mIoU values obtained from a single training and evaluation run for each configuration. Consequently, confidence intervals, repeated training experiments, and formal statistical significance tests were not performed. The reported differences between configurations should therefore be interpreted as observed performance differences under the evaluated experimental settings rather than as evidence of statistically significant superiority. Future work could incorporate repeated training runs and uncertainty quantification procedures to assess the robustness and statistical significance of performance differences across model configurations.

We propose extending the analysis period to more recent years (2020–2025) to evaluate the model’s response to intensifying climate change and vector expansion. However, this extension is currently limited by the partial availability of updated climatic, environmental, and sociodemographic data for these periods. Future efforts should prioritize completing and harmonizing these datasets for consistent temporal comparisons. It is also relevant to explore hybrid architectures–such as U-Net++ combined with Transformer modules–and interpretability techniques (e.g., Grad-CAM, SHAP) to better analyze the spatial contribution of each variable. Integrating these models into operational environments could strengthen epidemiological surveillance capacity and guide public health policies based on territorial evidence.

## Data Availability

The dataset used in this study is publicly available and can be found on GitHub: https://github.com/juanzamora28/dengue-colombia-unetpp.
